# The complement system in lipid-mediated pathologies

**DOI:** 10.3389/fimmu.2024.1511886

**Published:** 2024-11-20

**Authors:** Lejla Alic, Kristina Dendinovic, Nikolina Papac-Milicevic

**Affiliations:** ^1^ Department of Medical Biochemistry, Faculty of Medicine, University of Sarajevo, Sarajevo, Bosnia and Herzegovina; ^2^ Department of Neurophysiology and Neuropharmacology, Center for Physiology and Pharmacology, Medical University of Vienna, Vienna, Austria

**Keywords:** innate immunity, complement, lipid metabolism, lipid-mediated pathologies, complement system

## Abstract

The complement system, a coordinator and facilitator of the innate immune response, plays an essential role in maintaining host homeostasis. It promotes clearance of pathogen- and danger-associated molecular patterns, regulates adaptive immunity, and can modify various metabolic processes such as energy expenditure, lipid metabolism, and glucose homeostasis. In this review, we will focus on the intricate interplay between complement components and lipid metabolism. More precisely, we will display how alterations in the activation and regulation of the complement system affect pathological outcome in lipid-associated diseases, such as atherosclerosis, obesity, metabolic syndrome, age-related macular degeneration, and metabolic dysfunction-associated steatotic liver disease. In addition to that, we will present and evaluate underlying complement-mediated physiological mechanisms, observed both *in vitro* and *in vivo*. Our manuscript will demonstrate the clinical significance of the complement system as a bridging figure between innate immunity and lipid homeostasis.

## Introduction

Living organisms require immune homeostasis - a balance between the immune tolerance to self and immunogenicity to exogenous challenges deleterious to the host. This equilibrium is achieved through coordinated interplay between tissues with proteins and cells of the immune system. In contrast to pathogen-induced inflammation, metabolic changes caused by abnormal amounts of nutrients lead to sterile low-grade inflammation (1). This kind of inflammation can be initiated within various organs, and if not resolved by immune system action, it can drive disease development.

One of the essential energy sources, lipids, are fundamental for building cell structures, cellular signaling, and the generation of physiologically active compounds. They affect immunity in a bidirectional manner, e.g., through the balance between pro- and anti-inflammatory lipid-derived mediators modulating cellular and humoral immune response by shaping repertoires of immune cells, circulating antibodies and complement system components. At the same time, the immune system can steer lipid metabolism and determine the fate of lipid derivatives affecting the general metabolic homeostasis. Next to native lipids, modified lipids and lipoproteins are increasingly recognized as drivers in many cellular and immune processes and disease pathogenesis such as atherosclerosis, metabolic dysfunction-associated steatotic liver disease (MASLD), etc. (1–3).

Lipid-mediated pathologies comprise a broad spectrum of diseases in which abnormal lipid metabolism, signaling and storage affect various organs and systems. The most prevalent are obesity, atherosclerosis, type 2 diabetes mellitus (T2DM), age-related macular degeneration (AMD), and MASLD (4). This group of non-communicable diseases reduces the quality of life and is responsible for 20 million deaths annually (https://www.who.int/news-room/fact-sheets/detail/noncommunicable-diseases). Several factors, including genetics, diet, and lifestyle contribute to these complex conditions. The diagnosis is usually established by anthropometric measurements, biochemical, genetic, liver function tests, inflammatory markers, and imaging techniques.

In this mini-review, we will report recent knowledge on the interaction between lipids, lipid metabolism within tissues and the complement system.

## Complement system – an overview

The complement system, a humoral part of innate immunity, is a network of proteolytic cascades exerting its function in extracellular and intracellular fluids and on cellular surfaces. Most of its constituents are synthesized in the liver, whereas some are produced in immune or non-immune cells. It is an ancient defense system consisting of pattern recognition receptors (PRRs) and regulatory proteins, organized into three different pathways: classical (CP), lectin (LP) and alternative (AP). The CP is initiated by binding of its PRR - C1q, to antigen/antibody complexes, some pentraxins, apoptotic bodies and amyloid fibrils. Furthermore, repeating carbohydrates or acetylated residues, as well as aberrant glycocalyx patterns, engage recognition by PRRs of the LP (5–7). In contrast to that, the AP has a continuous low level of activation on self and non-self surfaces by the tick-over mechanism (8). Recently it was shown that complement can be activated in a non-canonical manner, by certain proteases from coagulation and fibrinolysis pathways (9–12). Cascades of all complement pathways conduct and control deposition of the central effector molecule C3 (13). Once deposited, and if not inactivated, C3 can guide the recruitment and formation of C5-convertase and terminal complement complex (C5b-C9) with a lytic function (8). Moreover, C3 and C5 cleavage generates anaphylatoxins that can drive chemotaxis and activation of immune cells, further propagating damage and inflammation (14, 15). Due to their strong auto-damaging potential, complement cascades are controlled by multiple regulators and inhibitors present in the fluid phase and on host cells (7, 16).

Novel evidence demonstrated that complement acts intracellularly. The intracellular complement, complosome, plays a role in cellular responses to the environment or the homeostatic balance maintenance, by regulating many cellular functions, e.g., cell metabolism, autophagy, survival, signaling, response to infections, and efferocytosis capacity (17–21).

Finally, the complement system functions are versatile: it eliminates pathogens and altered self-structures, coordinates innate and adaptive immune responses, controls tissue reorganization, instructs clearance of metabolic waste, and responds to metabolic alternations on intra- and extracellular levels (7, 17, 22).

## The complement system in atherosclerosis

Atherosclerosis is a chronic inflammatory disease characterized by the deposition and oxidation of low-density lipoprotein (LDL) particles in the vessel wall, followed by immune cell infiltration, leading to the formation of fatty streaks that can progress to plaques. If untreated, plaques become larger, more fibrous, calcified and prone to rupture. According to WHO, atherosclerosis is a major cause of mortality worldwide and is responsible for most myocardial infarctions (MI), strokes, and peripheral artery disease (PAD) (23).

The relevance of the complement system in atherosclerosis was demonstrated in the 1970s (24). This was followed by detection of complement proteins C3, C1q, C4, C9, C-reactive protein (CRP), C5b-C9, CD55, CD35, C3aR1, C5aR1, factor B (FB), factor H (FH), C1-inhibitor (C1-INH), C4-binding protein (C4BP), as well as active degradation products of some, within atherosclerotic plaques (25–32). Various clinical or genome-wide association studies (GWAS), demonstrated that components of the LP and CP have both proatherogenic and atheroprotective effects. More precisely, ficolin-1 and -2, pentraxin 3 (PTX3), mannan-binding lectin serine protease 2 (MASP2) and MASP3 have predictive value towards adverse cardiovascular events (33–35). The absence of mannan-binding lectin (MBL) predisposes to atherogenesis, but there are some controversies over its serum level effect on atherosclerotic cardiovascular disease (ACVD) (36–41). *C1Q* has been identified as a risk gene; in advanced lesions its local production is higher, but clear mechanisms of how its serum levels affect disease outcome are unknown (42–49). Predisposition for MI or cerebrovascular episodes was seen in hereditary *C2* deficiency (50). Also, elevated levels of C4 have been associated with cardiovascular disease (CVD) or diabetic stroke, independently of traditional risk factors (51–55). In contrast to CP or LP components, elevated C3 levels correlated with classical risk factors and worsened CVD outcomes (56–63). Similarly, components of the C5b-C9 complex have a strong association with pathogenicity (60, 64–69). Serum levels of C4BP can be predictive of the severity of the PAD (70, 71). Although AP is proatherogenic, there is no conclusion about the effect of *CFH* polymorphisms on coronary heart disease (CHD) (72–76). On the contrary, plasma concentrations of factor H-related protein 1 (FHR1) were elevated in patients with ACVD and correlated with the expression of the inflammation markers (77).

The mechanistic role of complement in atherosclerosis was explored *in vitro* and *in vivo*. High-fat diet (HFD) feeding led to elevated amounts of circulating lipids and increased levels of circulating C3, C4 and C1q (78, 79). Oxidatively modified LDL (OxLDL), located within the vessel, presents various damage-associated molecular patterns (DAMPs) on its surface, which are recognized by natural IgM antibodies, CRP, C1q, MBL, C3a, FH, FHR1, FHR3, FHR5 or scavenger receptors on macrophages (1, 2, 80–86). Due to its damage potential, if not neutralized by the immune system, OxLDL can activate endothelial cells. It alters their phenotype to procoagulatory and induces their secretion of C3a, C5a and other chemokines. This further propagates endothelial distress and activates immune response, partially through C3a/C3aR, C5a/C5aR axis, or by deposition of sublytic C5b-C9 (87–92). Consequently, monocytes are recruited to the intima, where they become foam cells (23). Macrophage uptake of OxLDL, cholesterol efflux and foam cell transformation is affected by PTX3, C3a, C1q, factor D (FD), FH and MBL (81, 84, 93–97). If the amount of engulfed lipids is too excessive, cholesterol crystals (CCs) build up and trigger NLR family pyrin domain containing 3 protein (NLRP3) activation, resulting in macrophage death. At this stage, IgM, C1q, MBL-A, MBL-C, and C3b play a protective role – guiding their removal of dying cells by macrophages, employing complement receptor 3 (CR3) and V-set and immunoglobulin domain containing 4 protein (VSIG4) receptors (98–102). Next to it, a balance between intracellular C3 activation and repression by FH controls the efferocytosis rate of lesional macrophages and affects necrotic core formation (19). The impaired clearance rate of lipid-overloaded macrophages leads to the release of DAMPs resulting in the recruitment of additional immune cells and generation of C3a and C5a, propagating inflammation, and smooth muscle cell expansion (103). This leads to the necrotic core formation filled with CCs, cellular debris, monocytes/macrophages rich in tissue factor and erythrocytes. Cholesterol crystals are recognized by C1q, C3c, ficolin-2, MBL, PTX3, and CRP. When cleared by CR3-rich monocytes, opsonized CCs activate the inflammasome in a C5a-dependent manner and enhance IL-1β secretion (104–108). Later, it was clarified that the metabolic switch required for IL-1β production by macrophages requires mitochondrial C5aR1 ligation generated by cell-intrinsic C5a (109). Furthermore, C5a makes plaques unstable and prone to rupture by affecting the senescence and death rate of smooth muscle cells (110, 111). Upon plaque rupture, released CCs activate complement and drive thrombus formation (112).

The presence of components of the C5b-C9 complex within lesions is shown to be proatherogenic *in vivo*. This observation was further supported by the finding that the absence of CD59, a C5b-C9 inhibitor, accelerated advanced atherosclerosis (113, 114). The previously mentioned proatherogenic effect of C5a was further confirmed by findings that inhibition of surface-expressed C5aR1 and deficiency of C5aR2 resulted in smaller lesions using animal models (111, 115–117).

The discrepancy in complement inhibition effectiveness between human trials and animal studies in atherosclerosis highlights the need for further research on the complement system function to develop novel therapeutic strategies (118–120).

## The complement system in metabolic syndrome

Metabolic syndrome (MetS) represents a cluster of several disorders, including insulin resistance (IR), obesity, dyslipidemia, hypertension and hyperglycemia. It contributes to the development of ACVD and T2DM (121–124). Even though increased C3 and C4 levels have been associated with the risk of developing MetS, metabolic alterations such as IR, obesity, inflammation, and neurohormonal dysfunction are pivotal initiators of this pathogenic cascade (123, 125–130).

*Insulin resistance* is characterized by the loss of sensitivity to insulin within insulin-dependent tissues such as adipocytes, muscles and liver. Observational studies have shown that C4 levels are associated with the homeostasis model assessment (HOMA) index, the parameter for IR (131). Although C1qA deficiency protects from HFD-induced IR, conflicting findings for the involvement of CP have been reported (132–135). In contrast to CP, LP is predominantly protective. Independently of multiple metabolic features, MBL correlated with insulin sensitivity and its levels were low among obese individuals (136–139). Additionally, low ficolin-3 was independently associated with IR and predicted type 2 diabetes mellitus (T2DM) (140). Baseline C3 levels and level changes correlated with HOMA, multiple organ IR and T2DM independently of obesity, metabolism- and inflammation-related risk factors (131, 141–149). Mechanistically, C3 influence on IR may be linked to the activity of C3a and C5a and their receptors. Studies in mice show that lack of C3aR or C3 can increase insulin sensitivity, although human studies did not find a connection between C3a and IR or T2DM (147, 150, 151). Furthermore, *in vivo* data on the role of C3adesArg – acylation stimulating protein (ASP) in IR are inconsistent (152, 153). In humans, higher ASP levels correlate with increased IR through altered lipid and glucose metabolism (154). Downstream proteins of the AP have been negatively correlated with insulin sensitivity. Weight loss and treatment of IR with rosiglitazone decreased FH concentrations in plasma, although conflicting results were found in SLE patients (126, 155). Additionally, the association between properdin, FH and Bb with HOMA was observed (156). However, data on the role of properdin obtained *in vivo* differ from human ones, as properdin deficiency did not affect insulin-mediated glucose uptake (157). Mechanistic data confirm the relevance of FB in MetS, as *Cfb*-/- mice exhibit increased insulin sensitivity and decreased inflammation (158). For more downstream components, *in vitro* and *in vivo* data have shown C5aR1 to contribute to IR development (159, 160). Furthermore, the role of C5b-C9 complex in IR is still unclear. For instance, it does not correlate with IR nor influence the incidence of T2DM (147). However, in chronic heart failure, a positive correlation with HOMA-IR, fasting glucose and insulin level was seen (161).

*Obesity* is a low-grade inflammatory disease defined by excessive fat accumulation in visceral and subcutaneous fat depots (BMI≥30kg/m^2^) (162, 163). It is driven by lifestyle, genetic, environmental and cultural factors and is considered endocrine and metabolic disease (162, 164–166). Excessive nutrient intake or low energy expenditure causes lipid accumulation, leading to adipocyte hypertrophy or hyperplasia, consequently driving inflammation that further exacerbates obesity and associated health issues (166–168). Adipose tissue produces many complement proteins including C3, FB, FH, CR1, C1q, C1r, C1s and properdin, and is the predominant source of key players in adipose tissue biology such as ASP, FD, and adiponectin (155, 169–173). In obesity, serum levels of C3, FB, FH and factor I (FI), but not FD, were elevated when compared to normal weight controls (174, 175). Similarly, analysis of BMI-discordant monozygotic obese twin pairs demonstrated that levels of FHR5, C4, C1qA, C1-INH, MASP1, FH, FI, C3 and C8 were elevated in a twin with higher BMI (176). Moreover, visceral and subcutaneous adipose tissue of a heavier twin had increased expressions of C1, C2, C3, FB, FI, properdin, FH, FHR2, C3aR, C5aR1, VISIG4, CD59, in contrast to FD and components of the C5b-C9 (177). In most rodent models of obesity, decreases in FD levels, induction of C1q, and inconsistency in overproduction and secretion of C3 or FB by adipose tissue were seen (170, 173, 178–180). Different production rate of certain complement proteins between subcutaneous and visceral fat of obese subjects, or between dissimilar stages of adipocyte maturation was reported in mice and men (172, 181–185). Within adipose tissue, by binding to the C5L2 receptor, ASP stimulates TG synthesis, increases glucose transport through GLUT1 and GLUT4, fractional free fatty acids re-esterification and inhibits lipolysis (186–194). In line with this, C3-deficient mice have a reduction in fat mass and are resistant to diet-induced obesity. Exogenous ASP administration to *C3*-/- animals on a standard diet led to a weight increase of a fat pad (193, 195–199). Overexpression of FB in preadipocytes boosts their lipid accumulation and maturation (185, 200). Additionally, through C3a/C3aR axis, FD regulates glucose uptake, increases TG synthesis and inhibits lipolysis (169, 172, 201–203). However, animals deficient in FD had no abnormality in development or body weight (204). Another adipokine similar to C1q is adiponectin, with an anti-inflammatory and anti-fibrotic function. It enhances insulin sensitivity and is downregulated in obesity (205–208).

*Diabetes mellitus* is the dysregulation of blood glucose levels due to insufficient insulin secretion by pancreatic beta cells, insensitivity of peripheral tissues to insulin, or a combination of both. Type 2 DM, an inflammatory disease, represents 90% of newly diagnosed cases. It is related to obesity and multiple metabolic disturbances, e.g., IR leading to hyperinsulinemia, beta cell exhaustion and finally insulin insufficiency (209). The complement system was shown to have critical metabolic functions within the beta cells. It affects insulin secretion, substrate and metabolite processing and regulates inflammatory processes within islets. Elevated C3 is associated with an increased risk of developing diabetes, independently of demographic, hereditary, metabolism- and inflammation-related factors (141, 147, 210). Interestingly, C3 was associated with insulin secretion, even after adjustment for insulin sensitivity index (211). Moreover, C3a, C3c and C3d correlated with T2DM, although for C3a these associations were attenuated after adjustment for confounding factors (147, 212, 213). Decreased levels of FD were observed in T2DM patients (174, 203). In one study, levels of properdin and soluble C5b-C9 were associated with a family history of T2DM, although the effect of C5b-C9 niveau was not confirmed in others (147, 156, 214, 215).

Since insulin infusion did not affect C3 expression within adipocytes, this implicated that C3 levels might affect insulin secretion *in vivo* (216). In line with this, increased C3 expression in T2DM pancreatic cells demonstrated that intracellular C3 has protective effects on islet beta cells in stress conditions, through interaction with ATG16L1 (18, 20). The highest expressed complement gene in human beta cells, *CD59*, was shown to control glucose-mediated insulin secretion (217–219). This effect is further promoted by ASP (220). On the contrary, beta cell FH suppresses insulin secretion via adrenomedullin (221). Additionally, C3a and C5b-C9 have been identified as potent inflammasome activators, suggesting their role in insulitis (222, 223). Interestingly, C3a and C5a generated by FD activity, and through the activity of their receptors, play a key role in adipose tissue-pancreas axis in murine models, by inducing insulin production and dampening beta cell death and dedifferentiation (201, 203, 224). Accordingly, C3aR and C5aR1 agonists improved glucose-dependent insulin production (225).

*Dyslipidemia* represents abnormal levels of lipids and lipoprotein particles in circulation and is a crucial risk factor for ACVD. It accounts for nearly 50% of deaths due to ischemic heart disease (226). Dyslipidemia is characterized by abnormal lipid levels, e.g., increased TGs, and decreased high-density lipoprotein (HDL). Many complement proteins have been shown to bind lipoprotein particles in plasma and are essential for their metabolic turnover. For instance, C3, acidic form of C4 (C4-A), basic form of C4 (C4-B), and C9 were detected on very low-density lipoprotein (VLDL) and LDL particles, while FHR3 appeared only on LDL (227, 228). Moreover, HDL was associated with C3, C4-A, C4-B, C9, vitronectin and clusterin in coronary artery disease and cholesterol ester protein transfer deficiency (229, 230). Additionally, FH, FD, properdin and MASP3 showed associations with lipoprotein particle concentration and size (228). Apolipoprotein E, a major protein component of lipoproteins, binds to FH, and C1q (97, 231, 232). In observational studies, levels of TGs, adverse lipoprotein subclass profile and enrichment in TGs in all lipoprotein subclasses are associated with higher levels of circulating C3; however, contradictory opinions were reported (129, 144, 216, 228, 233–238). Accordingly, ASP and TG levels correlate, although significance is lost when adjusted for waist/hip ratio and LDL size (195, 239). Elevated expression of C1S, C5aR1, CD59 and CD55 in subcutaneous adipose tissue was seen in patients with familial combined hyperlipidemia (240). Experimental data further underscores the significance of complement-lipid interaction. For instance, postprandial C3 and ASP secretion by adipocytes is shown to be stimulated by chylomicrons (190, 241). Additionally, male *C3*-/- mice (ASP deficient) display delayed postprandial clearance of TGs and increased fasting and postprandial free fatty acids levels (198). Properdin-deficient animals have increased fat accumulation on HFD, impaired postprandial TG clearance and decreased energy expenditure (157, 242).

As demonstrated above, the complement system controls insulin production and resistance, adipose tissue remodeling, and lipoprotein metabolism, consequently affecting chronic low-grade inflammation.

## The complement system in metabolic dysfunction-associated steatotic liver disease

The liver, a primary site for complement protein synthesis, is particularly susceptible to complement-mediated damage (243, 244). Dysregulation of the complement system can exacerbate liver inflammation and fibrosis (245).

Metabolic dysfunction-associated steatotic liver disease (MASLD) is characterized by excess fat accumulation in hepatocytes, without significant alcohol intake and is among the most commonly diagnosed liver disorders (246, 247). A progressive and inflammatory form of MASLD, known as metabolic dysfunction-associated steatohepatitis (MASH), features hepatic steatosis, inflammation, and fibrosis (248, 249). Predisposing factors for MASLD and MASH are components of MetS, oxidative stress, and lipid peroxidation. This condition can further advance to cirrhosis and hepatocellular carcinoma (250, 251).

A positive correlation between high C3 in serum and the prevalence and severity of MASLD was demonstrated in clinical studies (252–255). In addition to this, ASP, involved in adipocyte lipid metabolism, was increased in MASLD patients (255). Histological analyses demonstrated that in 74% of patients, cleaved C3b and C4d were deposited in liver tissue, with more than 50% of C3-positive livers also showing C1q and MBL deposits, and exhibiting C5b-C9 formation. Additionally, these components were more frequently detected in MASH, demonstrating the involvement of both CP and LP in liver inflammation (256). However, in some patients, C3 activation was not associated with C1q, MBL, or C4d deposition, suggesting that the AP may also play a role in complement activation in the MASLD. This was confirmed by the positive correlation of properdin and the C3c deposits with liver inflammation. Additionally, levels of FH were downregulated in MASH subjects (257). C3a and C5a were identified to promote hepatic inflammation by attracting immune cells (258). Additionally, blocking or removing C3aR and C5aR1 might offer protection against steatosis, fibrosing MASH, inflammation, and metabolic dysfunction (17, 259–261). However, while some evidence points to C3a/C3aR and C5a/C5aR1 as a potential therapeutic approach, clinical trials targeting the complement system are still lacking, making its effectiveness uncertain.

## The complement system and age-related macular degeneration

Age-related macular degeneration (AMD) is a chronic, inflammatory disease of the retina, and it is the most common cause of blindness in the elderly in developed countries (262). It is characterized by the accumulation of lipid-rich drusen between the Bruch membrane (BrM) and retinal pigment epithelium (RPE), resulting in degeneration of RPE, photoreceptors and consequently loss of vision. Joint action of aging, genetic and environmental factors play a significant role in the disease onset and progression (263).

Lipids within the retina are crucial players in the pathogenesis of AMD. Due to photo-oxidative stress, they are highly susceptible to lipid peroxidation and the generation of oxidized and reactive breakdown products (1, 264–267). These degradation products can initiate sterile inflammation and stimulate immune responses (1, 2, 266, 268–270).

Seminal studies discovered 52 variants with genome-wide association (GWA) significance, among which *CFH* variant rs1061170 (Y402H), and variants within *ARMS2-HTR1, C2-CFB-SKVI2L* and *C3* had the strongest associations, confirming the key role of complement in the AMD pathogenesis (271–273). Today, Y402H is recognized as the major susceptibility variant. Heterozygous carriers of the minor C allele have a 2-4-fold, and homozygous carriers have a 3-7-fold increased risk for developing AMD (274). Moreover, it is important to note that this variant also affects the splice variant of FH, FHL-1, a main regulator of AP within BrM and RPE (275–277). Y402H decreases the binding ability of FH to heparin sulfate, CRP and malondialdehyde (MDA), thereby affecting its availability to act and regulate AP on surfaces decorated with these ligands (85, 276, 278–281). A similar aftermath was observed for the rare variant R1210C located within the C-terminal part of FH (273, 282). Deletion of *CFHR3-1* genes decreases the risk of AMD development (283–285). Moreover, deleterious effects were reported for some variants within *CFI*, *C3* and *C9* (274, 286–296). On the other hand, loss-of-function variants within complement activators *C2* and *CFB* are mostly protective (293, 297, 298).

Accumulation of oxidative damage shown by intensive MDA staining in BrM and choroid which co-stained with FH, implicated the binding of FH to MDA-adducts. Additionally, FH promoted the generation of iC3b on MDA surfaces and suppressed the proinflammatory effects of MDA *in vitro*. Due to the weaker binding to MDA, Y402H carriers have diminished regulation of AP and attenuated anti-MDA inflammatory properties (85). This was supported in a murine model, expressing chimeric FH, containing a human Y402H variant (299). Loss of endogenous FH in RPE cells renders them more susceptible to oxidative stress and reduces their viability (300, 301). Furthermore, we have demonstrated that FHR1 and FHR3 bind to MDA and thereby compete with FH for it, affecting FH AP regulatory function. These findings mechanistically explain the protective properties of *CFHR3-1* deletion (80).

Next to AP, the relevance of locally produced C5b-C9, deposited in choriocapillaris during the photoreceptor outer segment recycling by RPE was reported (302–304). In the aging retina, recycling capabilities decrease, resulting in enhanced C5b-C9 formation, pronounced in the presence of AMD-predisposing *CFH* variants (303, 305). Apart from locally produced FHL-1 and C5b-C9, increased systemic C3d/C3 ratio and enhanced local C3d deposition are implicated in AMD (302, 306, 307).

Activation of the AP, dysfunctional FH, together with aging, oxidative damage and disturbed lipid metabolism, are identified as critical steps in the development and progression of AMD (7).

## Concluding remarks

**Figure 1:**
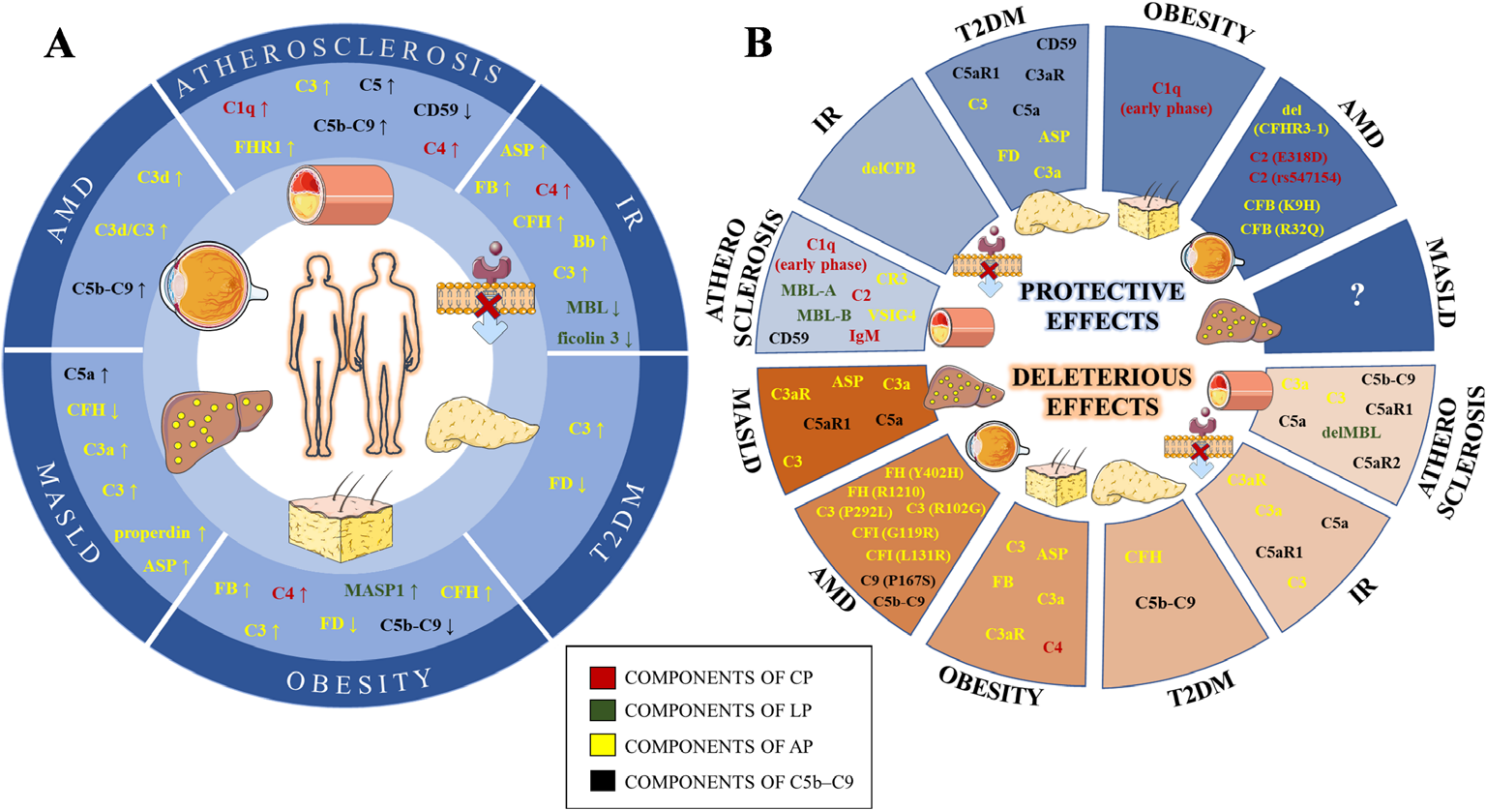
Schematic illustration of complement levels alterations **(A)** and their effects **(B)** in lipid-mediated pathologies. The components of the classical (CP), lectin (LP), alternative (AP) and terminal (C5b-C9) pathways are labeled in red, green, yellow and black, respectively. IR, Insulin resistance; T2DM, Type 2 diabetes mellitus; MASLD, Metabolic dysfunction-associated steatotic liver disease; AMD, Age-related macular degeneration; IgM, Immunoglobulin M; C1q, Complement Component 1q; C2, Complement Component 2; C4, Complement Component 4; MBL, Mannan binding lectin; MASP1, MBL Associated Serine Protease 1; C3, Complement Component 3; Asp, C3adesArg – acylation stimulating protein; FB, Factor B; FD, Factor D; FH, Factor H; FHR1, Factor H related protein 1; FI, Factor I; C3aR, Complement C3a Receptor; VSIG4, V-Set And Immunoglobulin Domain Containing 4; CR3, Complement Component Receptor 3; C5, Complement Component 5; C5aR1, Complement C5a Receptor 1; C5aR2, Complement C5a Receptor 2; C9, Complement Component 9; del, gene deletion; gene variants are given in the bracket after affected gene.

Experimental evidence presented in this review confirms that the functions of the complement system in lipid homeostasis are versatile (**Figure 1**). Certain complement proteins sense and control lipid homeostasis locally or systemically. However, in case of excessive lipid accumulation or oxidation, complement proteins, if unable to neutralize it, can also act as initiators or propagators of lipid-driven inflammation. Therefore, understanding all mechanisms involved in cross-talk between lipid metabolism and complement system should be of importance for developing better diagnostic or therapeutic approaches.
